# An Intradermal Inoculation Model of Scrub Typhus in Swiss CD-1 Mice Demonstrates More Rapid Dissemination of Virulent Strains of *Orientia tsutsugamushi*

**DOI:** 10.1371/journal.pone.0054570

**Published:** 2013-01-16

**Authors:** Piyanate Sunyakumthorn, Daniel H. Paris, Teik-Chye Chan, Margaret Jones, Alison Luce-Fedrow, Suchismita Chattopadhyay, Ju Jiang, Tippawan Anantatat, Gareth D. H. Turner, Nicholas P. J. Day, Allen L. Richards

**Affiliations:** 1 Viral and Rickettsial Diseases Department, Naval Medical Research Center, Silver Spring, Maryland, United States of America; 2 Mahidol Oxford Tropical Medicine Research Unit, Mahidol University, Bangkok, Thailand; 3 Nuffield Department of Clinical Laboratory Sciences, Oxford University, Oxford, United Kingdom; 4 Uniformed Services University of the Health Sciences, Bethesda, Maryland, United States of America; Kansas State University, United States of America

## Abstract

Scrub typhus is an important endemic disease of the Asia-Pacific region caused by *Orientia tsutsugamushi*. To develop an effective vaccine to prevent scrub typhus infection, a better understanding of the initial host-pathogen interaction is needed. The objective of this study was to investigate early bacterial dissemination in a CD-1 Swiss outbred mouse model after intradermal injection of *O. tsutsugamushi*. Three human pathogenic strains of *O. tsutsugamushi* (Karp, Gilliam, and Woods) were chosen to investigate the early infection characteristics associated with bacterial virulence. Tissue biopsies of the intradermal injection site and draining lymph nodes were examined using histology and immunohistochemistry to characterize bacterial dissemination, and correlated with quantitative real-time PCR for *O. tsutsugamushi* in blood and tissue from major organs. Soluble adhesion molecules were measured to examine cellular activation in response to infection. No eschar formation was seen at the inoculation site and no clinical disease developed within the 7 day period of observation. However, *O. tsutsugamushi* was localized at the injection site and in the draining lymph nodes by day 7 post inoculation. Evidence of leukocyte and endothelial activation was present by day 7 with significantly raised levels of sL-selectin, sICAM-1 and sVCAM-1. Infection with the Karp strain was associated with earlier and higher bacterial loads and more extensive dissemination in various tissues than the less pathogenic Gilliam and Woods strains. The bacterial loads of *O. tsutsugamushi* were highest in the lungs and spleens of mice inoculated with Karp and Gilliam, but not Woods strains. Strains of higher virulence resulted in more rapid systemic infection and dissemination in this model. The CD-1 mouse intradermal inoculation model demonstrates features relevant to early scrub typhus infection in humans, including the development of regional lymphadenopathy, leukocyte activation and distant organ dissemination after low-dose intradermal injection with *O. tsutsugamushi*.

## Introduction

Scrub typhus is an acute febrile disease caused by *Orientia tsutsugamushi*, a Gram-negative intracellular bacterium transmitted by larval trombiculid mites known as chiggers, which serve as both disease vectors and reservoirs for maintenance of *O. tsutsugamushi* in nature [1]. Scrub typhus is endemic in the Asian-Pacific region, where up to 28% of diagnosed febrile illnesses among hospitalized patients are due to scrub typhus, and case fatality rates can rise to 50% in untreated patients [2], [3], [4], [5], [6]. The features of *O. tsutsugamushi* strain virulence are understudied, and to completely understand host immune response, immunopathophysiology of severe disease and disparate virulence of various strains of *O. tsutsugamushi* a better animal model is required.

Various murine models of scrub typhus infection have been developed, and the CD-1 outbred Swiss mouse model is widely used to study host immune response and vaccine development [7], [8]. Outbred mice demonstrate broader and more heterogeneous immune responses that more accurately reflect the natural and vaccine induced immune responses as well as the associated immunopathophysiology in the human host [8], [9].

Intraperitoneal (IP) and intravenous (IV) injections are commonly used as routes of infection for *O. tsutsugamushi* in laboratory animals. However, they are not the natural route by which vertebrate hosts acquire *O. tsutsugamushi* infection in nature. Mice injected IP with *O. tsutsugamushi*, often in relatively high doses, demonstrate confined infection within the peritoneal cavity where *O. tsutsugamushi* continuously replicated, with macrophages playing a crucial role in controlling the bacterial burden [10], [11]. Additionally, splenomegaly and hepatic granulomas were observed despite the absence of *O. tsutsugamushi* in spleen and liver [12]. Conversely, in cynomolgus primates and humans, early dissemination of *O. tsutsugamushi* via hematogeneous and/or lymphatic system was accompanied by regional lymphadenopathy and subsequent systemic dissemination and onset of clinical manifestations [13], [14], [15].

In humans, intradermal (ID) inoculation of *O. tsutsugamushi* via chigger bites results in the formation of a localized pathological skin reaction termed an eschar in 7–97% of clinical cases and can be associated with local lymphadenopathy [15], [16], [17]. The degree to which the obligate intracellular *O. tsutsugamushi* infects cells and divides at the bite site, as opposed to taking a route of rapid dissemination to cause rapid systemic infection, remains unknown.

In order to mimic the natural course of infection via a chigger bite, we opted for a scrub typhus mouse model based on ID injection of *O. tsutsugamushi* at the dorsum of the external ear. The infectious ID dose from a chigger in nature is unknown, and the currently available murine ID50/LD50 doses are based on IP mouse data [7]. We therefore used 10^3^ MuID_50_ for ID inoculation as a standard dose used in scrub typhus vaccine studies [18]. The strains included in this study cause 90–100% (Karp), 50–60% (Gilliam) and 0% (Woods) mortality rate in CD-1 Swiss mice following IP inoculation [7]. The objective of this study was to investigate the early clinical features, lymph node involvement, and dissemination dynamics of these different *O. tsutsugamushi* strains of varying virulence.

## Materials and Methods

### Mice

Female CD-1 Swiss outbred mice from Charles River Laboratories, Inc (Wilmington, MA, USA) at 6–8 weeks of age were used for these studies. Mice were kept in animal biosafety level (ABSL)-2 laboratories prior to inoculation. Two days before inoculation, the mice were moved to an ABSL-3 laboratory to adapt to their new surroundings. The mice were then intradermally inoculated with 10^3^ MuID_50_ of one of three strains of *O. tsutsugamushi* Karp (Papua New Guinea), Gilliam (Burma) and Woods (Australia) into the dorsum of the right ear [7]. A liver and spleen homogenate of uninfected CD-1 Swiss mice was used as mock inoculum to inject negative control animals [7]. After inoculation, the clinical observation period focused on the local injection site and any signs of systemic disease for 7 days when all mice were euthanized. All animal research was performed under the approval of the Institutional Animal Care and Use Committee at the Naval Medical Research Center (Protocol Number: 11-IDD-34).

### Experimental Design

Three outbred CD-1 mice (Charles River Laboratory Inc., Wilmington, MA, USA) were injected intradermally per time point in the ear as previously described [19]. For all inoculations, mice were anesthetized using isofluorane (inhalation administration) and ketamine (IP injection). Intradermal injections of 10^3^ MuID_50_ of *O. tsutsugamushi* were performed at the right ear dorsum at a single site (5 µl of pre-titrated liver-spleen homogenate) using a 0.3 ml insulin syringe (Becton Dickinson, New Jersey, USA). Two mice were injected with mock inoculum (liver/spleen homogenate). Three different strains of *O. tsutsugamushi*, Karp (high virulence), Gilliam (intermediate virulence) and Woods (low virulence), were used to infect three groups of mice in two separate experiments. Following euthanasia, multiple samples from draining lymph node, liver, lung, kidney, spleen, whole blood, peritoneal cavity lavages (washing with sterile PBS), and skin biopsies from the ear were collected at 10 min, 45 min, 2 h, 6 h, 24 h, 3 d, and 7 d post inoculation (pi). Tissue blocks were either snap frozen in liquid nitrogen for subsequent quantitative real-time PCR (qPCR) or fixed in 10% neutral buffered formalin for histology and immunohistochemistry (IHC). Detection of *O. tsutsugamushi* at the injection site and draining lymph nodes (superficial parotid) was performed using IHC. Whole blood, liver, lung, kidney, spleen and peritoneal cavity lavages were analyzed for the presence of *O. tsutsugamushi* by *Orientia-*specific 47 kDa qPCR assay (see below). Centrifuged serum samples were used for analysis of anti-*O. tsutsugamushi* antibodies (IgM and IgG) and sCAMs (sE-selectin, sL-selectin, sICAM, and sVCAM) levels.

### Detection of anti-*O. tsutsugamushi* antibody responses

To determine the early development of murine IgG and IgM antibody responses against *O. tsutsugamushi* infection, serum samples collected from all time points were assessed by *Orientia*-specific ELISA assays as previously described without modification [20].

### Detection of soluble cell adhesion molecules (sCAMs)

Serum samples from all time points were assessed for sE-selectin, sL-selectin, sICAM-1 and sVCAM-1 by using ELISA kits (R & D Systems, Minneapolis, MN, USA) following manufacturer's instructions. Samples were assayed in duplicate. The plates were read for optical density at 465 nm (Vmax/Kinetic Microplate Reader, Molecular Devices, Sunnyvale, CA, USA). Quantity of sCAMs in mouse sera were compared at seven time points, and each time point includes two control mice (mock inoculated with uninfected liver spleen homogenate), and six mice each inoculated with *O. tsutsugamushi* Karp, Gilliam, or Woods strains.

### Immunohistochemical analyses

Skin biopsies were obtained from each mouse using 8-mm circular biopsy punch (Stiefel Laboratories Inc., Offenbach, Germany), and cut into half prior to fixation. Skin biopsies and draining lymph nodes were fixed in 10% neutral buffered formalin solution (Sigma, St. Louis, MO, USA) for at least 2 days, and then embedded into paraffin blocks. Paraffin-embedded tissues were sectioned at 4 μm. The sections were de-paraffinized with Citroclear (TCS Biosciences, Buckingham, UK) for 5 min twice, 100% ethanol for 2 min twice, and 50% ethanol for 2 min. De-paraffinized sections were then used to perform microwave-based antigen retrieval in Tris-EDTA buffer (pH 9) for 10 min. In order to localize *O. tsutsugamushi* in tissue sections, anti-*O. tsutsugamushi* mouse monoclonal antibody (isotype IgG2bκ) kindly provided by Ampai Tanganuchitcharnchai was utilized for immunoenzymatic and immunofluorescence staining as previously described [15].

Immunoenzymatic staining was performed using Novalink Polymer Detection System (Leica, Newcastle, UK), according to manufacturer's instruction with minor modification. Briefly, endogenous peroxidase activity was neutralized by Peroxidase block solution for 5 min, and tissue sections were incubated with anti-*O. tsutsugamushi* monoclonal antibody for 30 min. After washing, tissue sections were incubated with Post Primary Block solution for 30 min and Novolink polymer for 30 min. Peroxidase activity was developed using 3, 3′-diaminobenzidine substrate (DAB) chromogen. The slides were then counterstained with hematoxylin and mounted with mounting medium before microscopic observation.

For immunofluorescence staining, de-paraffinized tissue sections were incubated with anti-*O. tsutsugamushi* monoclonal antibody for 1 h. After washing, the slides were incubated with Alexa Flour 488 goat anti-mouse IgG_1_ antibody (Molecular Probes, Eugene, OR, USA) for 1 h. Then the tissue sections were mounted in Vectashield mounting medium with DAPI (4′, 6-diaminidimo-2-phenylindole) (Vector Laboratories, Burlingame, CA, USA) and observed using fluorescence microscope.

### Quantitation of *O. tsutsugamushi*

Determination and quantitation of *O. tsutsugamushi* after inoculation was performed using a previously described qPCR assay for the *Orientia*-specific 47 kDa gene [21]. Templates were based on genomic DNA extracted from whole blood, liver, lung, kidney, spleen and peritoneal cavity lavages using the DNeasy tissue kit (Qiagen, Valencia, CA, USA) according to the manufacturer's instructions. Fifty microliters of DNase RNase-free water were used to elute the isolated DNA. The mouse-specific probe and primers were designed from the single copy gene: mouse complement factor D (*cfd*) as follows: *cfd* probe; 5′ -5HEX-CTGGGTTGGAGGTGTCTGTGGT-BHQ2-3′, *cfd*_For492 primer; 5′-ACTGAGATCGCTTTTGGGTC-3′, and *cfd*_Rev599 primer; 5′-GGAGGGTAGGTGTATTGTAAGG-3′) were designed using Primer3 software. Briefly, a total reaction volume of 25 μl consisting of 1 μl of DNA template, 15 μl of Platinum PCR Supermix (Invitrogen, Carlsbad, CA, USA), 100 nM of each primer, 200 nM of probe, and DNase/RNase-free water. Thermocycler parameters included 50°C for 2 min and 95°C for 2 min followed by 45 cycles of a two-step amplification protocol of 95°C for 15 sec, and 60°C for 30 sec. All reactions were performed on a Rotor-Gene 3000 cycler (Corbett Research, Mort lake, NSW, Australia), “no template” negative controls were run with each reaction and plasmid DNA served for standard curves in serial dilutions from 10^6^ to 3copies/μl of 47 kDa protein and mouse *cfd* genes. Quantitation of 47 kDa protein gene was expressed per 10^7^ mouse cells and normalization of the *O. tsutsugamushi* concentration of inocula, was performed (Karp inoculum: 132 organisms/injection, Gilliam inoculum: 830 organisms/injection and Woods inoculum: 261 organisms/injection).

### Statistical analysis

Data of soluble cell adhesion molecules are expressed as median and inter-quartile range, and the significant differences were determined using two-way analysis of variance (ANOVA), Graphpad Prism 6 software. A *P* value of ≤0.05 was considered significant.

## Results

### Clinical observation and gross examination

**Figure 1:**
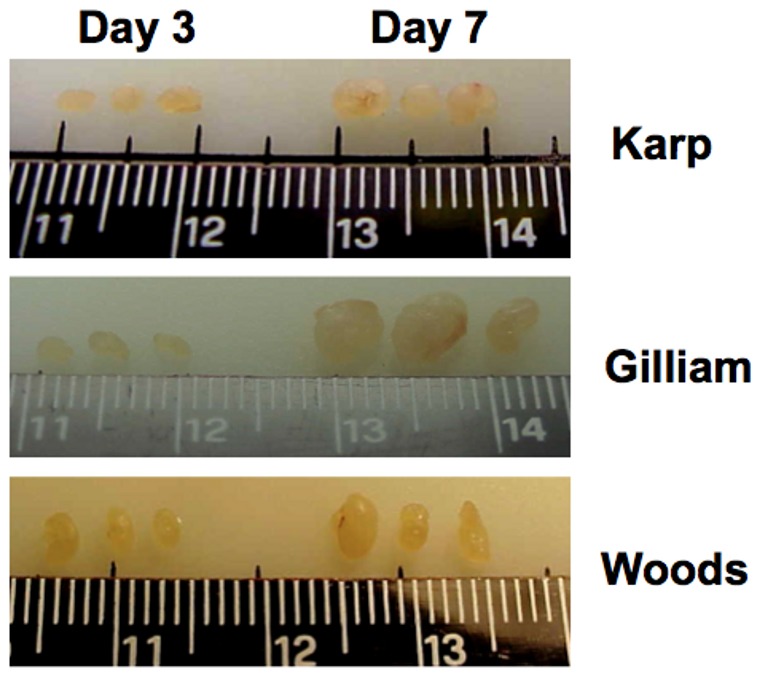
Macroscopic evidence of regional lymphadenopathy in *O. tsutsugamushi* inoculated mice. Gross examination of draining lymph nodes (right superficial parotid lymph node) from *O. tsutsugamushi* inoculated mice at day 3 pi (left) and day 7 pi (right) shows enlargement of lymph nodes at day 7. Top panel: Karp-inoculated mice, Middle panel: Gilliam-inoculated mice, Bottom panel: Woods-inoculated mice.

Three mice per strain per timepoint were examined in duplicate. All mice appeared healthy during the study period of 7 days with no clinical signs of disease or illness observed in any inoculated mice. There was no eschar formation or any observable induration or reaction at the injection site in any of the mice by day 7 pi. However, by day 7 pi all mice inoculated with one of the three strains of *O. tsutsugamushi* had developed regional lymphadenopathy in the superficial parotid lymph node as shown in Figure 1.

### Detection of anti-*O. tsutsugamushi* antibody responses

To determine the antibody responses against *O. tsutsugamushi*, serum samples from mice were assayed for the presence of *O. tsutsugamushi*-specific antibodies (total IgM and IgG). The control mouse sera (derived from 9 dpi IP-inoculated mice) indicated that antibodies against *O. tsutsugamushi* were detected in the positive controls; however, *O. tsutsugamushi* ID inoculated mice evaluated in this study did not mount a detectable antibody titer within the 7 day observation period.

### Detection of circulating soluble cell adhesion molecules

**Figure 2:**
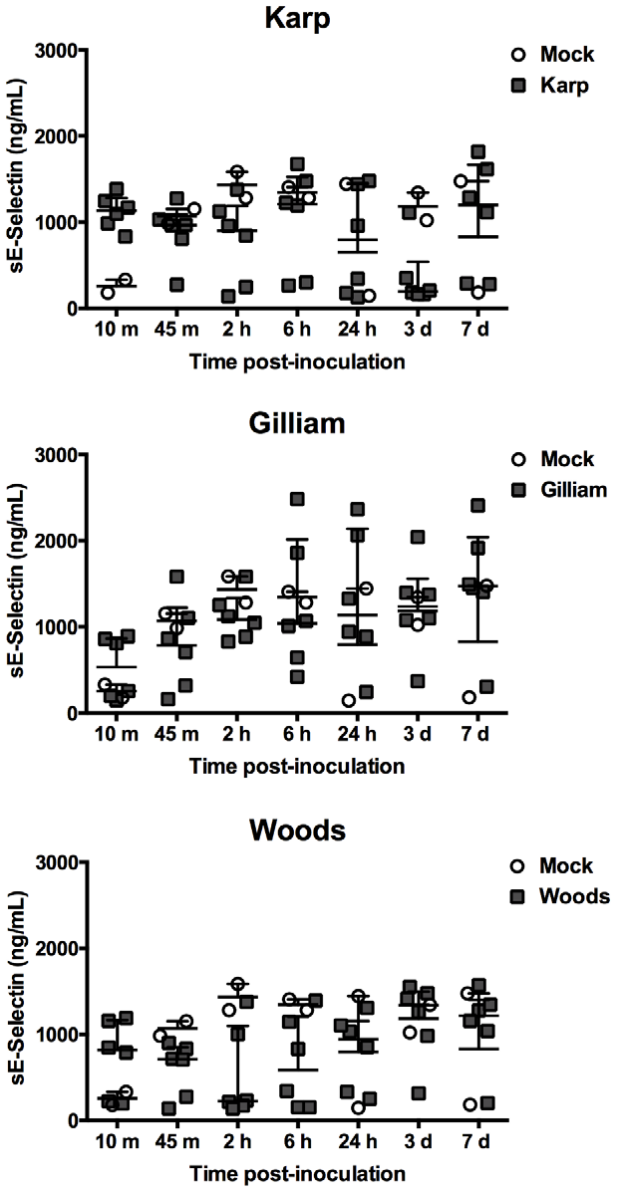
Serum levels of circulating soluble E-selectin. Dot plots demonstrate sE-selectin levels in inoculated mouse sera. (Mock inoculation; n = 2 per time point, *O. tsutsugamushi* inoculation; n = 6 per time point). Bars indicate median and error bars represent interquartile range.

**Figure 3:**
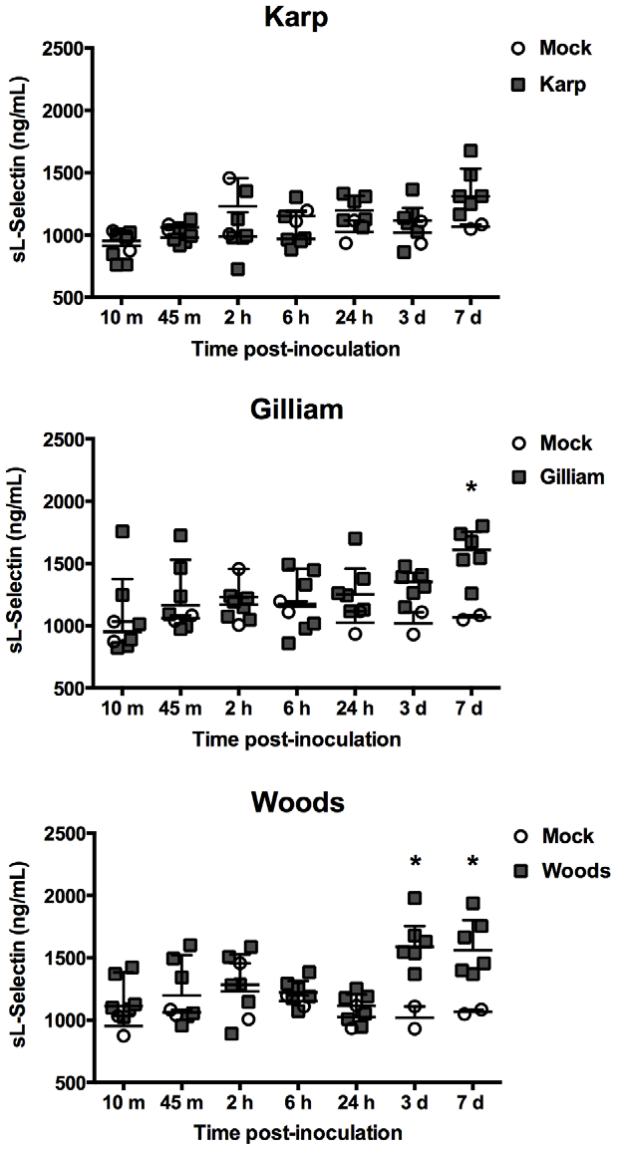
Serum levels of circulating soluble L-selectin. Dot plots demonstrate sL-selectin levels in inoculated mouse sera. (Mock inoculation; n = 2 per time point, *O. tsutsugamushi* inoculation; n = 6 per time point). Bars indicate median and error bars represent interquartile range. The asterisks indicate significant differences (*P*<0.05).

In order to investigate surrogate markers of endothelial and leukocyte activation following ID inoculation of *O. tsutsugamushi*, serum levels of circulating sE-selectin, sL-selectin, sICAM-1, and sVCAM-1 were measured at all time points. The mock- and *O. tsutsugamushi*-inoculated mice demonstrated no significant differences in the serum levels of sE-selectin at any time point (Figure 2). However, at day 3 pi, the levels of sL-selectin was significantly elevated by 59% in Woods-inoculated mice and by 49% in Gilliam- and Woods-inoculated mice at day 7 pi compared to mock-inoculated mice (Figure 3). No elevation of sL-selectin serum levels was observed in Karp-inoculated mice.

**Figure 4:**
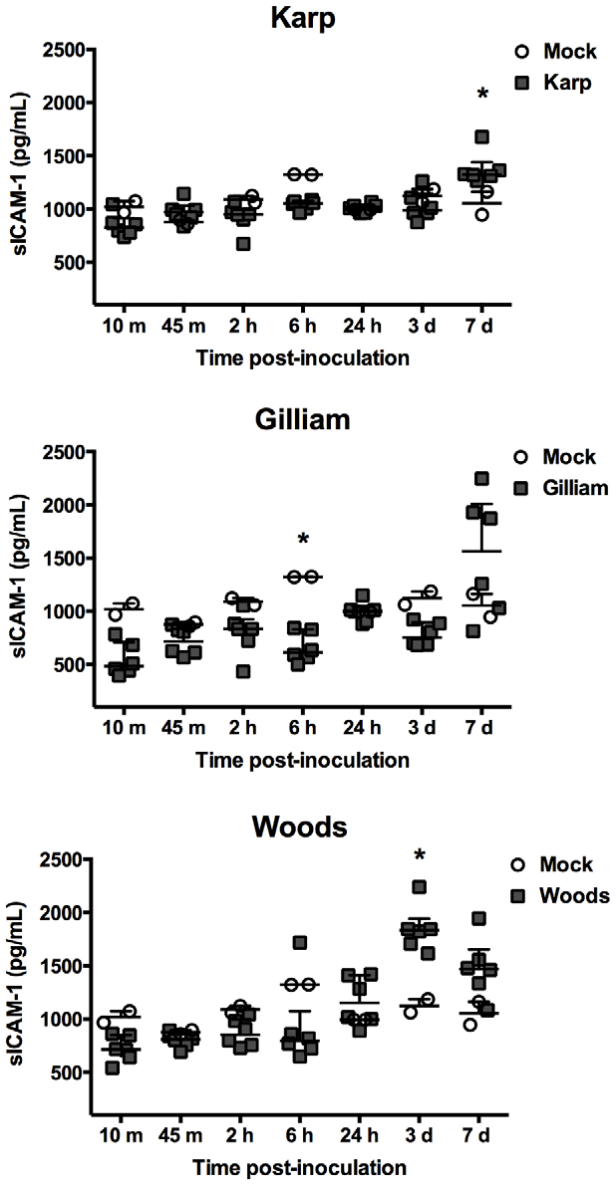
Serum levels of circulating soluble ICAM-1. Dot plots demonstrates ICAM-1 levels in inoculated mouse sera. (Mock inoculation; n = 2 per time point, *O. tsutsugamushi* inoculation; n = 6 per time point). Bars indicate median and error bars represent interquartile range. The asterisks indicate significant differences (*P*<0.05).

**Figure 5:**
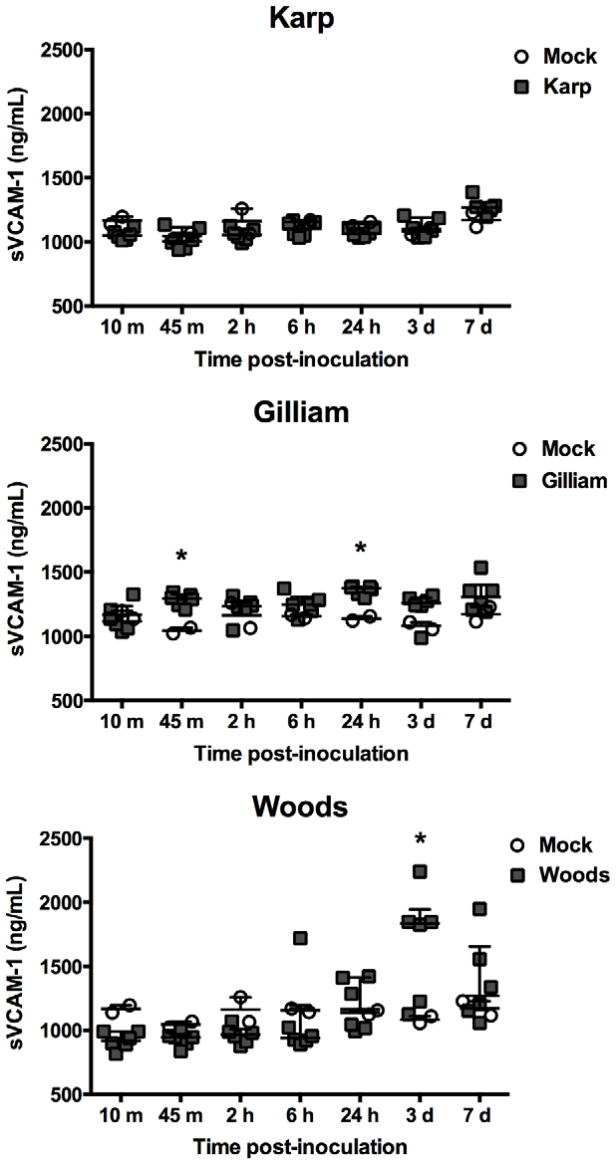
Serum levels of circulating soluble VCAM-1. Dot plots demonstratesVCAM-1 levels in inoculated mouse sera. (Mock inoculation; n = 2 per time point, *O. tsutsugamushi* inoculation; n = 6 per time point). Bars indicate median and error bars represent interquartile range. The asterisks indicate significant differences (*P*<0.05).

Increased serum level of sICAM-1 was seen by 30% in Karp-inoculated mice at day 7 pi and by 64% in Woods-inoculated mice on day 3 pi compared mock-inoculated mice (Figure 4). Similarly, sVCAM-1 levels were significantly increased by 55% at day 3 pi in Woods-inoculated mice, and in Gilliam-inoculated mice, 20% and 22% increase of sVCAM-1 levels was observed at 45 minutes and 24 hours pi, respectively (Figure 5).

### Immunohistochemical detection of *O. tsutsugamushi*

**Figure 6:**
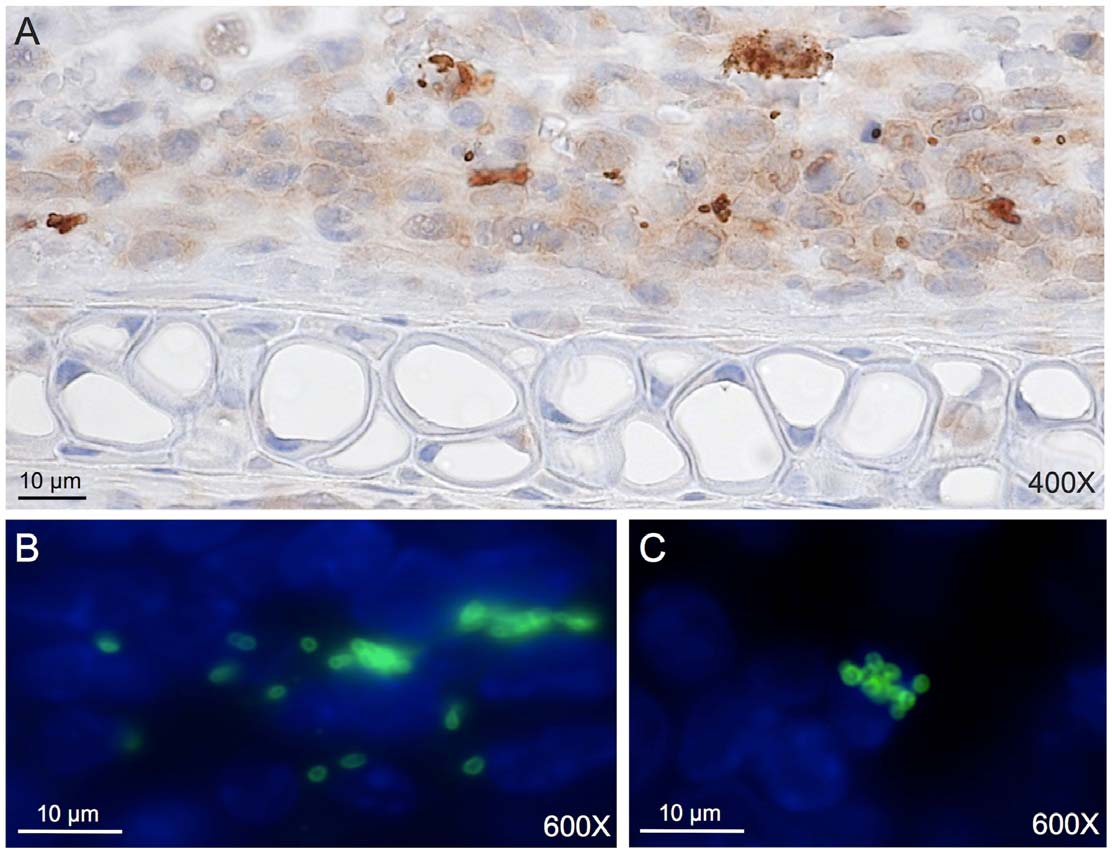
Detection of *O. tsutsugamushi* at the injection site (Panels A and B) and draining lymph node (Panel C) at 7 days post inoculation. In panel “A” *O. tsutsugamushi* in a mouse ear is demonstrated using peroxidase DAB reaction as brown colored dots (counterstain hematoxylin, original magnification x400). The immunofluorescence staining of *O. tsutsugamushi* is shown in panels “B” (mouse ear) and “C” (draining lymph node): *O. tsutsugamushi* with FITC (green) and DAPI nuclear counterstain in blue (original magnification x600).

To detect and localize *O. tsutsugamushi* organisms at the site of injection and the draining lymph nodes, immunoperoxidase and immunofluorescence staining was performed on tissue sections. At day 7 pi, all Karp-inoculated mice demonstrated a presence of *O. tsutsugamushi* at the injection site and the draining lymph node (Figure 6). Only 33% of Gilliam and Woods-inoculated mice showed *O. tsutsugamushi* at the injection site, and in draining lymph node *O. tsutsugamushi* was observed in 100% of Gilliam-inoculated mice and 66% of Woods-inoculated mice. Numerous *O. tsutsugamushi* organisms were observed in Karp-inoculated tissues compared to Gilliam- and Woods-inoculated tissues, and fewer organisms were found in Woods-inoculated tissues compared to Gilliam strain.

### Dissemination of *O. tsutsugamushi* Karp, Gilliam, and Woods strains

**Figure 7:**
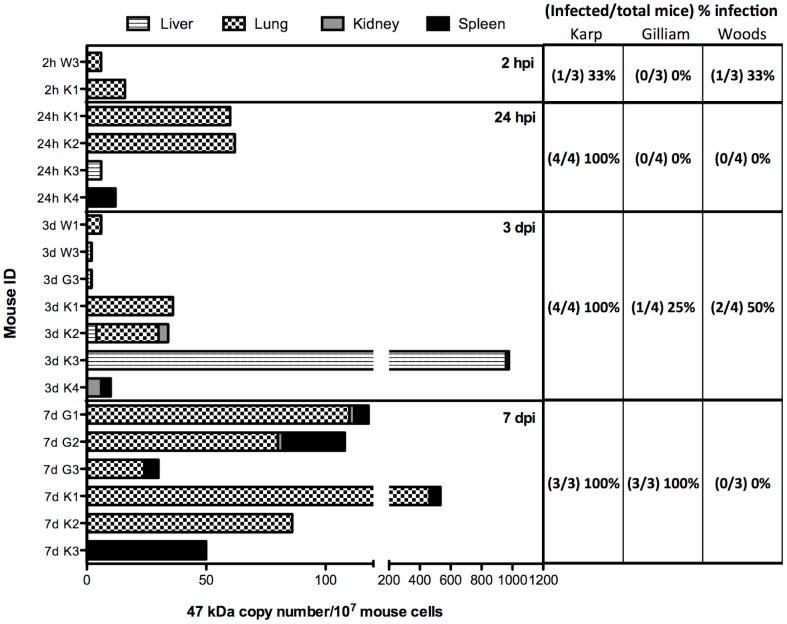
Bacterial load of *O. tsutsugamushi* in inoculated mouse tissues. *O. tsutsugamushi* copy numbers per 10^7^ mouse cells in mouse tissues (liver, lung, kidney, and spleen) at 2 hpi, 24 hpi, day 3 pi, and day 7 pi were quantitated using an *Orientia*-specific 47 kDa qPCR assay. The percentage of *O. tsutsugamushi* infected mice at each time point is shown in table. Abbreviations used: Karp (K), Gilliam (G), and Woods (W).

To determine the early dissemination of *O. tsutsugamushi* bacteria in mice after ID injection, samples of whole blood, lung, liver, kidney, spleen, and peritoneal cavity lavages were collected for bacterial quantitation using the *Orientia*-specific 47 kDa qPCR assay. *O. tsutsugamushi* was found in lung, liver, kidney and spleen samples but not in whole blood and peritoneal cavity lavage samples (Figure 7). Dissemination of Karp strain was rapid, as evidenced by infection of all Karp-inoculated mice by 24 hours pi, whereas it took 7 days for the Gilliam strain infected mice to show 100% *O. tsutsugamushi* infection, and this was not seen in Woods strain even by day 7 pi. There was an overall increase in bacterial copy numbers of *O. tsutsugamushi* in tissues over the 7 days observation period in Karp- and Gilliam-infected mice, which was not seen in Woods strain. In addition, the number of infected organs was higher in Karp-infected mice on day 3 pi than when less pathogenic strains were used. During the observation period, *O. tsutsugamushi* were found initially and predominantly in lung samples, and by day 7 pi increasingly in spleens as well (Figure 7).

## Discussion

A variety of animal models have been utilized to investigate the mechanisms of protective immunity and associated pathology in scrub typhus [18], [22]. The outbred mouse is more appropriate for vaccine evaluation studies [8], [23] whilst inbred mice strains allow investigation of more specific host humoral and cell-mediated immune responses and certain immunopathological mechanisms [11], [24], although various mouse strains show differing susceptibility to *O. tsutsugamushi* infection [8], [25], [26]. The CD-1 Swiss outbred mouse is the most commonly used laboratory animal model for scrub typhus vaccine response studies [8], [27], [28].

Studies on differential strain virulence are very limited [29], [30]. Previously published studies in mice have relied predominantly on the use of an IP injection model, which may not reflect the natural inoculation pathway and associated immune responses (i.e. intradermal deposition of *O. tsutsugamushi* via Leptotrombidium chiggers)[10]. The IP inoculation route results predominantly in infection of peritoneal macrophages and does not induce subsequent interactions with intradermal cells such as resident tissue macrophages and dendritic cells, which could orchestrate innate and adaptive immune responses and subsequent dissemination patterns.

Recently, a CD-1 Swiss outbred mouse model was reported after *O. tsutsugamushi* inoculation via chigger bite on the ear. The results demonstrated that inoculated CD-1 mice developed multiple clinical manifestations including hepatosplenomegaly and accumulation of peritoneal cavity fluid and died from infection at days 14–23 pi [28]. Although little is known about the initial development of *O. tsutsugamushi* in murine skin in the early stages of infection and the mechanisms of dissemination to cause systemic infection, this study was based on the skin as entry portal of infection. Compared to human scrub typhus, the IP inoculation mouse model failed to reflect an early course of natural infection, associated with a skin lesion or eschar, regional then systemic lymphadenopathy and subsequent systemic disease [4], [15].

In this study, we used ID injection to mimic chigger inoculation in its natural disease course, because the route and dose of inoculum could be controlled as they are factors likely to be critical in the subsequent clinical course, such as eschar development, local lymph node spread and dissemination [14]. The use of three *O. tsutsugamushi* strains (Karp, Gilliam, and Woods), with variable degrees of virulence previously defined in IP mouse models was designed to provide data on whether the early disease course was affected by bacterial virulence.

The inoculated CD-1 outbred mice developed regional lymphadenopathy and evidence of systemic leukocyte and endothelium activation, and the presence of *O. tsutsugamushi* was observed at the injection site and associated draining lymph nodes. However, no eschar formation was observed within the 7 days observation period. The Karp strain, which had been shown previously to have a higher virulence than Gilliam and Woods strains, was associated with earlier and more extensive dissemination to distant organs.

The lack of eschar formation observed at the injection site suggests that the local immune responses at the inoculation site of CD-1 Swiss outbred mice and humans are different. This may influence clinical course and response to infection. Literature suggests that the cynomolgus non-human primate model (*Macaca fascicularis*), is better suited for scrub typhus immunopathophysiological studies as intradermal inoculation of *O. tsutsugamushi* causes eschar formation and systemic disease similar to humans. However, the investigation of the innate immune response at the inoculation site of macaques compared to human requires more detailed characterization [22].

To investigate surrogate measures of cellular activation we measured serum levels of sCAMs, which included sE-selectin, sL-selectin, sICAM-1 and sVCAM-1. These sCAMs are endothelial leukocyte adhesion molecules expressed by cytokine-activated endothelial cells and/or leukocytes, and promote recruitment of leukocytes to sites of inflammation site. L-selectin also supports mononuclear cell migration to lymph nodes. They can serve as surrogate markers for endothelial and leukocyte activation and show differential upregulation in rickettsial diseases, as previously reported in human scrub and murine typhus patients [31]. This study demonstrated that mononuclear cell activation with elevated serum levels of sL-selectin was associated with scrub typhus. However, sE-selectin levels were raised as well and associated with the presence of eschar, lymphadenopathy and elevation of circulating leukocyte count.

Within the observation period of this study, raised levels of sE-selectin were observed in all groups, however no increase of sE-selectin levels over time was observed in *O. tsutsugamushi*-inoculated mice. This may be suggestive of increased background endothelial activation with early systemic endothelial activation in the early stages of *O. tsutsugamushi* infection in this murine model, despite evidence of infection both locally at the inoculation site and subsequently in multiple distant organs. However, to better understand the endothelial activation of scrub typhus the measurement of sE-selectin later in disease course is needed. Markers of leukocyte activation including sICAM-1, sVCAM-1 and more specifically sL-selectin were, on the other hand, significantly elevated in *O. tsutsugamushi*-inoculated mice at days 3-7 pi, similar to the rise seen in human cases [31]. The significant increase of L-selectin compared to E-selectin suggested that, similar to patients with acute scrub typhus, mononuclear cell activation is more prevalent in early *O. tsutsugamushi* infection in mice than endothelial activation.

In scrub typhus, bacterial dissemination precedes systemic infection in vertebrate hosts after ID inoculation [20]. We evaluated the rate and extent of dissemination of three *O. tsutsugamushi* strains with different mouse virulence; Karp (high-virulent strain), Gilliam (intermediate-virulent strain), and Woods (low-virulent strain) strains using *Orientia*-specific qPCR assay to assess bacterial burden in blood and different organs in a time course study. At 24 hpi, all Karp inoculated mice demonstrated systemic infection, and compared to Gilliam and Woods strains, Karp showed earlier and more extensive dissemination (Figure 7). Local dissemination of *O. tsutsugamushi* to draining lymph nodes was observed by immunohistochemical staining by day 7 pi, in all strains. Despite the limitations of detecting low-level blood-borne *Orientia* by PCR, the evidence of spread to distant organs (within day 2 pi for Karp) argues that there may be more than one pathway for dissemination in this early phase of infection (i.e. lymphatic and hematogenous pathways). Following inoculation and presumably invasion of host cells in the dermis, some bacteria can spread gradually to local lymph nodes within a week, whereas others disseminate more rapidly to distant organs (e.g. hematogenously), however the cellular tropism underlying these alternative routes remain unclear.

Although it was observed that lymphadenopathy and sL-selectin levels were more pronounced in the lower virulence subgroups further immunological investigations will reveal if differences and dynamics of induced immune responses are associated with bacterial virulence in mice. Further, while in this study the same dose previously used for IP injection by Chan et al. [7] was used via an ID route, it has to be considered that the bacterial dose of an ID inoculum will affect transmission dynamics and host responses, thus a dose effect experiment will be of high importance. However, little is known about the natural dose inoculated by chiggers and how the saliva of chiggers modulates both sides of the host-pathogen interactions.

In conclusion, CD-1 outbred mice developed regional lymphadenopathy and leukocyte and endothelium activation after low dose intradermal injection with different *O. tsutsugamushi* (Karp, Gilliam, and Woods strains). Local dissemination to peripheral lymph nodes and distant organs was observed; however, no eschar formation was seen at the inoculation site. Spread to distant organs, predominantly lung and spleen was rapid, but mediated by low numbers of organisms below the sensitivity of qPCR assay in blood to detect circulating rickettsemia. Karp strain inoculation was associated with more extensive and rapid systemic infection than lower virulence Gilliam and Woods strains. Further investigations into the host innate immune response at the inoculation site will require the use of a more suitable animal model, to determine how a protective immune response can be achieved against challenge by *O. tsutsugamushi* via the natural ID route of infection.
